# Circulating MicroRNAs Regulating DNA Damage Response and Responsiveness to Cisplatin in the Prognosis of Patients with Non-Small Cell Lung Cancer Treated with First-Line Platinum Chemotherapy

**DOI:** 10.3390/cancers12051282

**Published:** 2020-05-19

**Authors:** Chara Papadaki, Alexia Monastirioti, Konstantinos Rounis, Dimitrios Makrakis, Konstantinos Kalbakis, Christoforos Nikolaou, Dimitrios Mavroudis, Sofia Agelaki

**Affiliations:** 1 Laboratory of Translational Oncology, School of Medicine, University of Crete, Heraklion, Vassilika Vouton, 71003 Crete, Greece; chapapadak@uoc.gr (C.P.); monasal91@gmail.com (A.M.); mavroudis@uoc.gr (D.M.); 2 Department of Medical Oncology, University General Hospital of Heraklion, Vassilika Vouton, 71110 Crete, Greece; rounis@gmail.com (K.R.); dim.ph.makrakis@gmail.com (D.M.); konkalbakis@yahoo.gr (K.K.); 3 Department of Biology, University of Crete, Heraklion, Vassilika Vouton, 70013 Crete, Greece; nikolaou@uoc.gr; 4 Institute of Molecular Biology and Biotechnology, Foundation of Research and Technology, Heraklion, Vassilika Vouton, 70013 Crete, Greece

**Keywords:** circulating miRNAs, NSCLC, platinum-based chemotherapy, DNA damage response, hypoxia

## Abstract

The expression of microRNA (miR)-21, miR-128, miR-155, and miR-181a involved in DNA damage response (DDR) and tumor responsiveness to platinum was assessed by RT-qPCR in the plasma of patients with non-small cell lung cancer (NSCLC; n = 128) obtained prior to initiation of first-line platinum chemotherapy. U6 small nuclear RNA (snRNA) was used for normalization, and fold change of each miRNA expression relative to the expression in healthy controls was calculated by the 2^−ΔΔCt^ method. MicroRNA expression levels were correlated with patients’ outcomes. Integrated function and pathway enrichment analysis was performed to identify putative target genes. MiR-128, miR-155, and miR-181a expressions were higher in patients compared to healthy donors. MiRNA expression was not associated with response to treatment. High miR-128 and miR-155 were correlated with shorter overall survival (OS), whereas performance status (PS) 2 and high miR-128 independently predicted for decreased OS. In the squamous (SqCC) subgroup (n = 41), besides miR-128 and miR-155, high miR-21 and miR-181a expressions were also associated with worse survival and high miR-155 independently predicted for shorter OS. No associations of miRNA expression with clinical outcomes were observed in patients with non-SqCC (n = 87). Integrated function and pathway analysis on miRNA targets revealed significant enrichments in hypoxia-related pathways. Our study shows for the first time that plasma miR-128 and miR-155 hold independent prognostic implications in NSCLC patients treated with platinum-based chemotherapy possibly related to their involvement in tumor response to hypoxia. Further studies are needed to investigate the potential functional role of these miRNAs in an effort to exploit their therapeutic potential.

## 1. Introduction

Despite recent progress in diagnosis and therapy, non-small cell lung cancer (NSCLC) remains a deadly disease with survival rates having only minimally improved in the last decades [1]. Even though novel targeted therapies and immune checkpoint inhibitors (ICIs) have been introduced in daily clinical practice, platinum-based chemotherapy remains the mainstay of treatment in early as well as in metastatic disease [2]. Chemotherapy efficacy though is significantly hampered by the development of primary or acquired resistance in most of the patients [3].

The main mechanism of action of cisplatin and its analogues is the formation of DNA adducts followed by intra- and interstrand cross-links that block DNA replication and induce DNA damage [4]. DNA damage then results in the activation of Ataxia telangiectasia mutated (ATM) and Ataxia telangiectasia and Rad 3-related (ATR) kinases that phosphorylate a wide range of downstream targets within a complex network of signaling pathways operating in cell-cycle arrest, DNA repair, and apoptosis [5]. Activation of the apoptotic machinery results in cell death, whereas successful lesion repair leads to cell survival and drug resistance [6]. Reported mechanisms of cisplatin resistance include decreased intracellular accumulation of cis-diamminedichloridoplatinum (II) (CDDP), increased detoxification systems, impaired apoptotic signalling after DNA damage and DNA repair, or tolerance of the damage [7].

MicroRNAs (miRNAs), a class of small noncoding RNAs, regulate gene expression by posttranscriptional repression of their mRNA targets [8]. Cancer is associated with global alteration of miRNA expression patterns, where miRNAs have been shown to exert either oncogenic or tumor suppressive effects depending on the context [9]. Each miRNA is implicated in a wide array of pathological processes through the regulation of multiple gene targets, and inversely, each mRNA can be targeted by multiple miRNAs [10]. Conceivably, the expression pattern of a small number of miRNAs may reflect extensive alterations of gene expression networks involving hundreds of messenger RNAs [11]. Bioinformatics provide the opportunity for systemic analysis of pathways and biological processes that are specifically regulated by multiple dysregulated miRNAs, thus resulting in meaningful insights in the mechanisms underlying different steps of cancer cell function and tumor progression [12].

Accumulating evidence suggests that critical genes involved in DNA repair are epigenetically regulated by miRNAs [13]. Furthermore, DNA damage response results in the transcriptional or posttranscriptional modulation of miRNAs [13] which regulate DNA damage response according to the nature and intensity of DNA damage [14]. In addition, miRNAs have been associated with response or resistance to various types of cancer drugs including cisplatin [15].

Thus, miR-21 modulates cell cycle progression and DNA damage checkpoint activation via cell division cycle 25 phosphatase (Cdc25A), which is involved in cell cycle arrest in response to DNA damage [16]. MiR-21 has been also associated with resistance of NSCLC cells to cisplatin through phosphatase and tensin homolog (PTEN) targeting and inhibition of apoptosis [17], whereas its deletion sensitized cells to DNA-damaging chemotherapy [18]. MiR-128 represses E2F transcription factor 5 (E2F5), leading to the transcriptional induction of cyclin-dependent kinase inhibitor 1A (*p21^waf1^*). In turn, p21 protein is localized to the cytoplasm, where it exerts antiapoptotic function in response to cisplatin [19]. MiR-155 silencing enhances the sensitivity of A549 lung cancer cells to cisplatin by activating the initiator caspase-9 through apoptotic peptidase activating factor 1 (Apaf-1) [20]. In addition, miR-155 has been shown to target tumor protein P53 (TP53) through a miR-155/TP53 negative feedback mechanism, ultimately leading to cisplatin resistance [21]. Upregulation of miR-181a results in BCL2-associated apoptosis regulator (Bax) oligomerization and caspase activation, thus enhancing cisplatin cytotoxicity [22]. In addition, miR-181a/b negatively regulates DNA damage response by impairing the expression and activity of the stress-sensor kinase ATM [23].

MiRNAs are detected in the plasma and other biological fluids in various physiologic and pathologic conditions including cancer [24]. There is evidence suggesting that circulating miRNAs promote tumorigenesis and participate in cancer invasion, metastasis, and angiogenesis by delivering signals in distant sites [25]. In addition, few studies show their function in modulating chemosensitivity [26]. Accordingly, it is considered that circulating miRNAs could serve as important biomarkers reflecting the dynamic processes occurring during tumor evolution [27] and predicting therapeutic response in cancer [28]. According to the above, we hypothesized that miRNAs involved in the regulation of cisplatin cytotoxicity may have a role as circulating biomarkers for the prediction of therapeutic efficacy of platinum-based chemotherapy in NSCLC. Thus, in the current report, we investigated the clinical significances of miR-21, miR-128, miR-155, and miR-181a expressions in the plasma of patients with NSCLC treated with first-line platinum-based doublets. As a great number of protein-coding mRNA targets have been reported for the four miRNAs under study, we performed a detailed bioinformatics analysis in order to narrow down and to prioritize the associated genes and pathways in the context of our study.

## 2. Results

### 2.1. Study Design and Patients Characteristics

**Figure 1:**
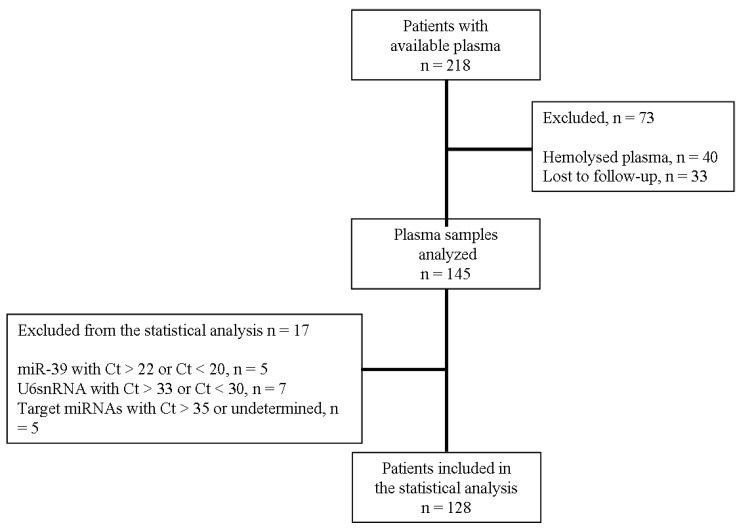
Flow chart of the study. Ct, cycle threshold.

The flow chart of the study and patients’ characteristics are shown in Figure 1 and Table 1, respectively. Patients with recurrent or metastatic NSCLC (n = 218) and available plasma obtained before the initiation of first-line platinum-based chemotherapy were enrolled in the current study; a total of 128 patients was included in the current analysis (Figure 1). Patients were predominantly males (87%), 96% had stage IV disease, and 68% had non-SqCC NSCLC; median age was 65 years (range, 37–88 years).

### 2.2. miRNA Expression and Statistical Correlations

**Figure 2:**
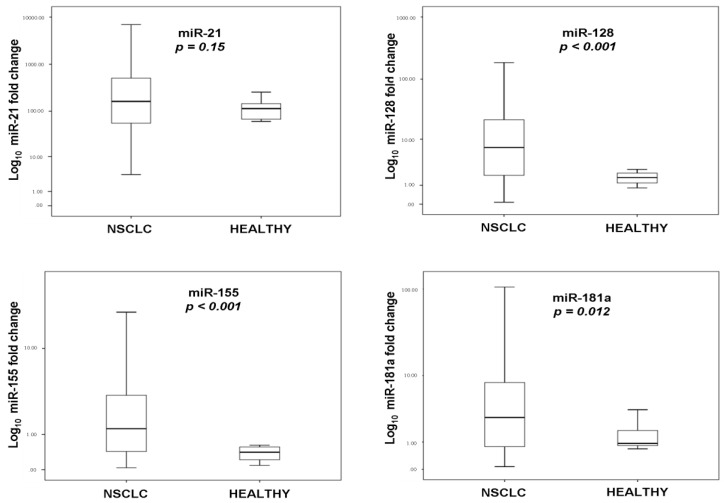
Fold change of four miRNAs expression in plasma of non-small cell lung cancer (NSCLC) (n = 128) patients treated with first-line chemotherapy and healthy donors (n = 19). Expression levels of four miRNAs relative to U6 snRNA was assessed by the 2^−ΔCt^ method. Mann–Whitney test was used to determine statistically significant differences, and the results are displayed on box plots. Horizontal line depicts median, whereas the length of the boxes is the interquartile range that represents values between the 75th and 25th percentiles of individual fold change expression values. Relative expression values on the *y*-axis are plotted on a log_10_ scale. *p* values are shown.

The fold change of plasma miRNAs expression relative to U6 snRNA was compared in NSCLC patients (n = 128) and in healthy donors (n = 19). The relative expression levels of miR-128, miR-155, and miR-181 were higher in NSCLC patients compared to healthy donors (Mann–Whitney test; *p* < 0.001, *p* < 0.001, and *p* = 0.012, respectively) (Figure 2).

No association was observed between miRNAs expression and patients’ characteristics. Moreover, miRNA expression did not differ among patients with SqCC and non-SqCC histology (Mann–Whitney tests, *p* > 0.05). However, strong correlations were revealed between the expression of the aforementioned miRNAs in the patients’ group. Specifically, miR-21 expression was strongly correlated to miR-128 (Spearman’s Rho: 0.853; *p* < 0.001), miR-155 (Spearman’s Rho: 0.829; *p* < 0.001), and miR-181a expressions (Spearman’s Rho: 0.896; *p* < 0.001) (Table 2). In addition, a strong correlation was observed between miR-128 and miR-155 (Spearman’s Rho: 0.855; *p* < 0.001) and miR-181a (Spearman’s Rho: 0.929; *p* < 0.001) expressions as well as between miR-155 and miR-181a expressions (Spearman’s Rho: 0.886; *p* < 0.001) (Table 2).

### 2.3. miRNA Expression and Clinical Outcomes

**Figure 3:**
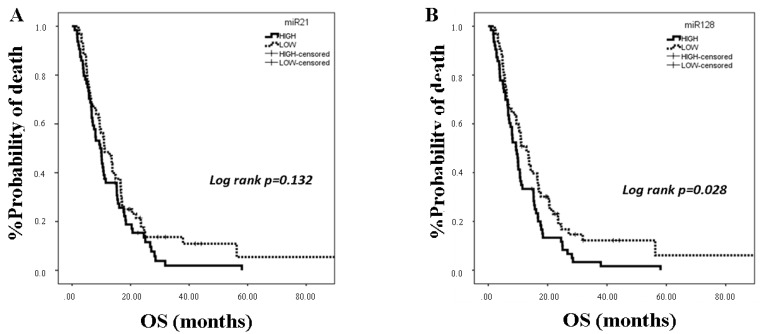

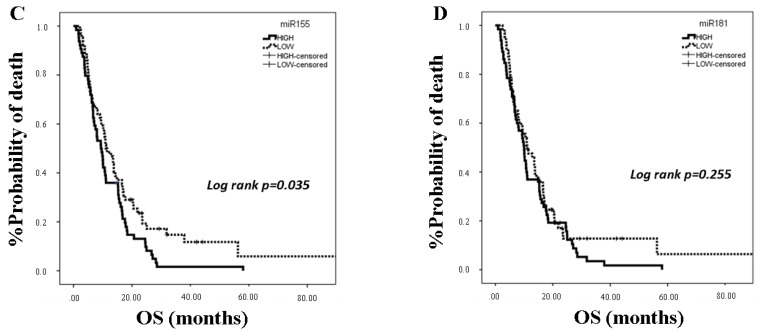
Kaplan Meier analysis for overall survival (OS) according to miRNA expression in the plasma of NSCLC (n = 128). Patients were classified into high and low expression groups according to the median value of each miRNA. OS in patients with high or low expression of miR-21 (**A**), miR-128 (**B**), miR-155 (**C**), and miR-181a (**D**). Curves were compared using the log rank test. *p* values are shown.

Twenty-six % of patients experienced objective response, 39% had stable disease, and 35% progressed (assessment was performed according to The Response Evaluation Criteria in Solid Tumors (RECIST) 1.1 criteria). The median progression free survival (PFS) was 4.8 months (95% CI: 3.94–5.66), and median OS was 10.2 months (95% CI: 8.93–11.47). The association of miRNA expression with clinical outcomes was evaluated by classifying patients as having high or low expression according to the median value for each miRNA. No correlations were observed between miRNA expression and response to chemotherapy (chi-squared tests, *p* > 0.05). The expression levels of miRNAs were not correlated with PFS (*p* > 0.05). However, patients with high expressions of miR-128 and miR-155 had shorter OS compared to patients with low expression (9.37 vs. 12.83; *p* = 0.028 and 9.37 vs. 11.13; *p* = 0.035, respectively) (Figure 3B,C). No associations were evident among miR-21 and miR-181a expressions and clinical outcome (Figure 3A,D).

Univariate Cox regression analysis revealed that PS 2 (*p* = 0.001), the presence of 2 or more metastatic sites (*p* = 0.006), high miR-128 (*p* = 0.03), and high miR-155 (*p* = 0.036) were associated with worse OS (Table 3). Multivariate Cox regression analysis confirmed that PS 2 and high miR-128 were independent predictors for worse OS (*p* = 0.003 and *p* = 0.026, respectively) (Table 3).

### 2.4. miRNA Expression and Clinical Outcome According to Lung Cancer Subtype

**Figure 4:**
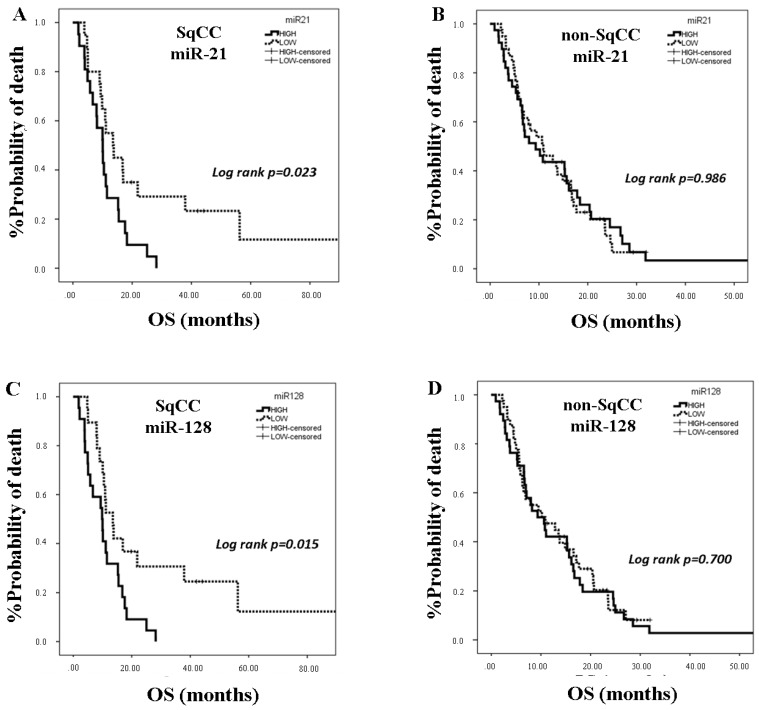

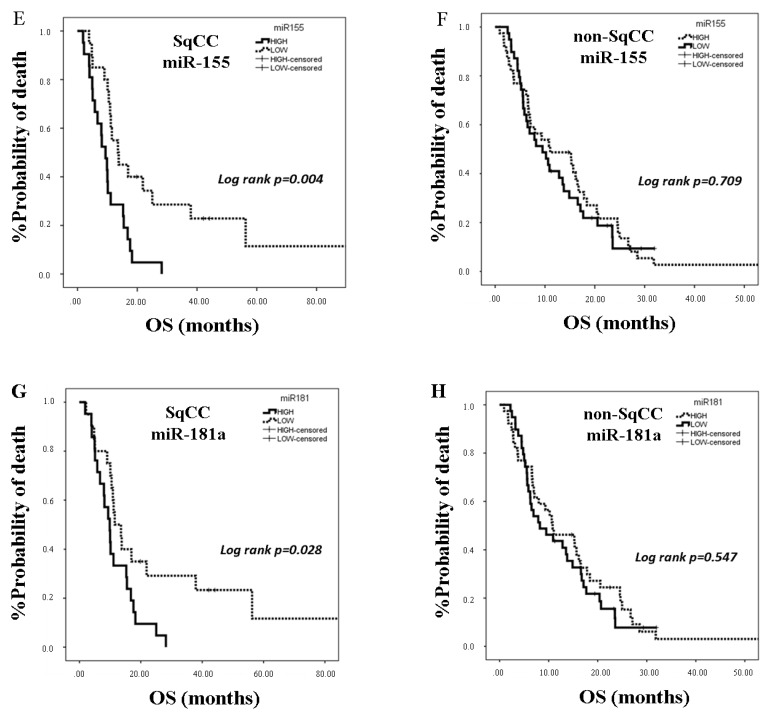
Kaplan Meier analysis for overall survival (OS) according to miRNAs expression in the plasma of squamous cell carcinoma (SqCC; n = 41) and non-squamous cell carcinoma (non-SqCC; n = 87) NSCLC patients. Patients were classified into high and low expression groups according to the median value of each miRNA. OS in patients with high or low expression of miR-21 (**A**,**B**), miR-128 (**C**,**D**), miR-155 (**E**,**F**), and miR-181a (**G**,**H**). Curves were compared using the log rank test. *p* values are shown.

Patients characteristics for SqCC (n = 41) and non-SqCC (n = 87) are shown in Table 1. No differences in miRNA expression were revealed among patients with SqCC and non-SqCC. In the SqCC subgroup, no correlations were observed between miRNA expression and clinical characteristics or response to treatment. Median PFS and OS were 5.53 months (95% CI: 3.27–7.79) and 10.9 months (95% CI: 9.57–12.23), respectively. Patients were divided into high and low expression levels according to the median value of each miRNA in order to find associations with clinical outcomes. No correlations were observed between miRNA expression and PFS (*p* > 0.05). However, patients with high expressions of miR-21, miR-128, miR-155, and miR-181a had shorter OS compared to patients with low expression (10.07 vs. 13.53, *p* = 0.023; 9.87 vs. 13.53, *p* = 0.015; 9.37 vs. 13.53, *p* = 0.004 and 9.87 vs. 11.5, *p* = 0.028, respectively) (Figure 4A,C,E,G).

In univariate Cox regression analysis, PS 2 was associated with shorter OS (*p* = 0.022) (Table 4). Regarding miRNAs expression, high miR-21, miR-128, miR155, and miR-181a were associated with worse OS (*p* = 0.026, *p* = 0.012, *p* = 0.004, and *p* = 0.03, respectively) (Table 4). In multivariate Cox regression analysis, only miR-155 high expression was an independent predictor for worse OS (*p* = 0.004) (Table 4).

In non-SqCC group, no correlations were observed between miRNA expression and clinical characteristics or response to treatment. Furthermore, no associations were evident between miRNA expression and PFS or OS (Figure 4B,D,F,H).

### 2.5. miRNA Target and Pathway Enrichment Analysis

**Figure 5:**
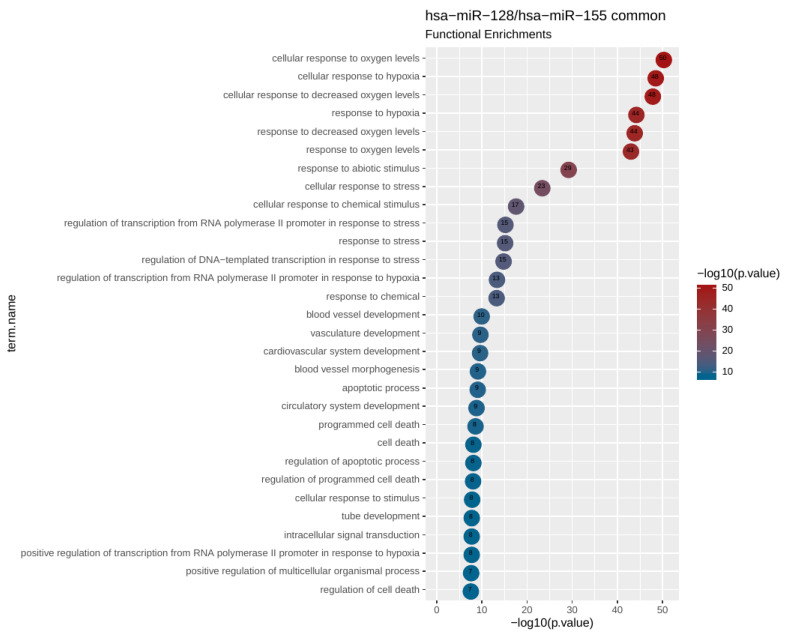
Functional enrichment plot of the subset of 26 overlapping genes that are associated with cellular response to hypoxia and are common targets to miR-128 and miR-155. Hypoxia functions are expectedly enriched alongside a number of pathways related to vasculature development, cell death, and angiogenesis. Each bubble represents a term and includes the *p*-value.

**Figure 6:**
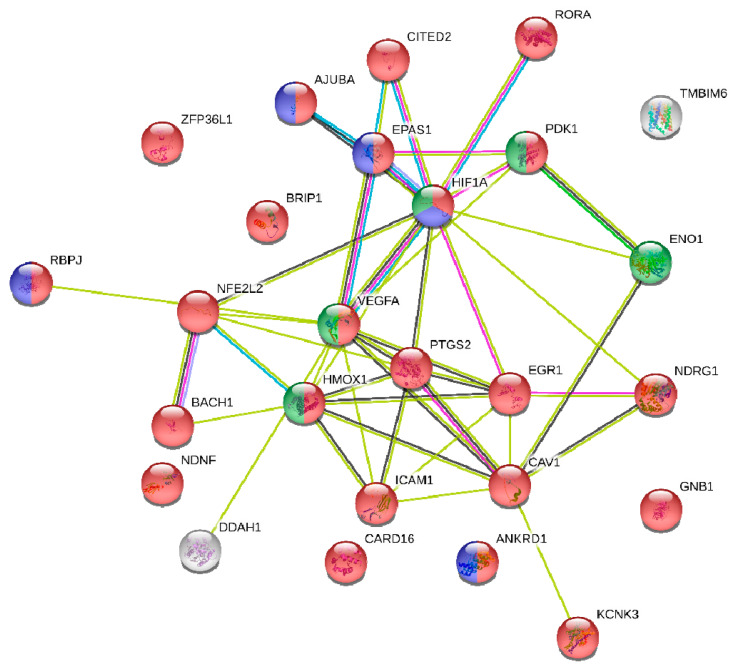
Protein–protein interaction (PPI) network of 26 common miR-128 and miR-155 targets that are associated with hypoxic response as obtained from the STRING Database (STRING-DB; https://string-db.org/). Members of the core pathway are coloured in red, transcriptional regulators are in blue, and members of the hypoxia inducible factor-1 a (HIF-1a) signalling pathway are in green. Connecting edges in black denote co-expression and show a strong network clique, which includes HIF-1a, vascular endothelial factor A (VEGFA), prostagladinG/H synthase and cyclooxygenase (PTGS2 (COX2)), intercellular adhesion molecule 1 (ICAM), heme oxygenase 1 (HMOX1), and endothelial PAS domain-containing protein 1 (EPAS1).

Pathway analysis of mRNA targets for both miR-128 and miR-155 revealed significant enrichments for a number of pathways and functions associated with hypoxia. When analyzing the set of common protein-coding targets for the two miRNAs, cellular response to hypoxia was ranked as the most enriched functional category with significant difference from the rest (Table 5). This prompted us to further analyze the particular gene set, comprising 26 genes that were associated to hypoxia and at the same time predicted to be targeted by both miR-128 and miR-155 (Table 6). We thus performed a focused functional analysis on this gene set alone. The top 30 most enriched functional terms are shown in Figure 5. These include, besides hypoxia-related functions, pathways that are associated with vasculature development, angiogenesis, apoptosis, and cell death. A more detailed analysis of these 26 genes at the level of protein–protein interaction (PPI) was performed with the use of STRING database (STRING-DB; https://string-db.org/). The resulting PPI network is highly enriched in transcriptional regulatory proteins, which is indicative of a dense regulatory circuit affected by (and probably also including) the miRNAs under study (Figure 6). The structure of the network also suggests strong connectivity between a set of core proteins, including hypoxia inducible factor 1 subunit alpha (HIF-1a), vascular endothelial growth factor alpha (VEGFa), and cyclooxygenase 2 (COX2) (prostaglandin-endoperoxide synthase 2, PTGS2). Connections between these proteins as well as intercellular adhesion molecule 1 (ICAM1), heme oxygenase 1 (HMOX1), and endothelial PAS domain protein 1 (EPAS1) are supported by gene co-expression and bibliographic data.

## 3. Discussion

Over the last few years, circulating miRNAs have been recognized as potential diagnostic and prognostic biomarkers in patients with cancer [25]. In the present study, we investigated the clinical relevance of plasma miR-21, miR-128, miR-155, and miR-181a in NSCLC patients treated with first-line platinum-based doublets and demonstrated that high expressions of circulating miR-128 and miR-155 were associated with shorter OS. In the SqCC subgroup, patients with high miR-21, miR-128, miR-155, and miR-181 had decreased OS compared to those with low expression values. Importantly, high miR-128 independently predicted for worse OS in the whole population whereas high miR-155 emerged as an independent predictor for worse OS in squamous NSCLC. Bioinformatics analysis of mRNA targets revealed interesting associations related to potential molecular mechanisms of response or resistance to treatment regulated by these miRNAs.

MiR-128, via Drosha ribonuclease III (Drosha) and Dicer ribonuclease III (Dicer) targeting, is a key regulator of the malignant phenotype in lung cancer cells by promoting epithelial to mesenchymal transition (EMT) and cell migration [30]. In addition, miR-128-3p has been reported as the only miRNA significantly upregulated in NSCLC as compared with normal tissue in The Cancer Genome Atlas (TCGA) lung cancer data sets [31]. Similarly, in a large-scale analysis of miRNA profiles, miR-128-3p emerged amongst the most commonly upregulated miRs in lung cancer tissue [32]. Importantly, in a cohort of NSCLC patients treated with cisplatin-based chemotherapy, high tumor miR-128 independently predicted for worse outcome [31]. Furthermore, miR-128 expression in the whole blood could distinguish early lung cancer patients from healthy donors [33]. We here demonstrate for the first time that high plasma miR-128 is an independent prognostic factor for worse survival in NSCLC patients treated with first-line platinum doublets.

MiR-155 is an oncomir with regulatory roles in tumor growth, EMT, metastasis, apoptosis, and response to chemotherapy [21,34]. MiR-155, along with miR-21, is among the most frequently amplified miRNA genes in NSCLC [35]. Unique miRNA profiling studies demonstrated that lung adenocarcinoma or SqCC presented higher miR-155 expression levels compared to normal tissues and suggested that its oncogenic role [34] is possibly related to direct inhibition of tumor suppressors, PTEN, and the suppressors of cytokine signalling 1 and 6 (SOCS1 and SOCS6) [36]. High tumor miR-155 has been demonstrated as an independent prognostic factor for poor overall survival in patients with lung adenocarcinoma [34] or SqCC [37]. Moreover, miR-155 was previously demonstrated to be differentially expressed in the plasma of patients with early NSCLC compared to healthy donors and was included in the plasma signature of 3 miRNAs associated with increased risk for progression in resected lung adenocarcinoma [38]. Furthermore, in a small cohort of NSCLC patients, Gao et al. showed that serum levels of miR-155 in combination with the tumor markers carcinoembryonic antigen (CEA) and CA-125 increased the efficiency for the early diagnosis of lung adenocarcinoma [39]. Our results further support the association of miR-155 with poor prognosis in NSCLC, and they show for the first time that plasma miR-155 represents an independent poor prognostic indicator in the squamous subtype.

MiR-21 and miR-155 share nearly 30% of their predicted targets [36]. In a previous report, both miRNAs expressed in tumor tissue, promoted NSCLC progression, and predicted recurrence and unfavorable survival of patients with stage I-III NSCLC [36,40]. Moreover, plasma miR-21 and miR-155 were amongst the miRNAs with the highest performance to discriminate patients with SqCC among early stage NSCLC patients [41]. In addition, high serum miR-21 expression was correlated with shorter survival in patients with early stage NSCLC [42]. We here show for the first time that high plasma miR-21 was associated with shorter OS in the SqCC subtype in patients with advanced stages of the disease and that circulating miR-21 and miR-155 expression levels were strongly correlated, potentially explaining the independent prognostic role for miR-155 only.

MiR-181 family members operate as tumor suppressors in lung cancer in contrast to their role in other tumor types [43]. Low miR-181a expression in NSCLC tissue was significantly correlated with poor patient survival [43]. We here demonstrate for the first time that plasma miR-181a expression levels are prognostic in NSCLC patients treated with first-line chemotherapy. Our findings regarding the association of high plasma miR-181a with shorter OS in the squamous patient subgroup potentially suggest a differential biological role of circulating miR-181a in SqCC NSCLC. 

It should be noted here that, although the investigated miRNAs were selected based on their implications in DNA damage response and/or the modulation of response to the cytotoxic effects of cisplatin, no associations were found between their plasma levels and response rates or PFS with platinum doublets. These observations imply that they may have a role in determining prognosis in patients treated with platinum-based doublets. Additional studies are required to further define their prognostic relevance in NSCLC patients treated with other modalities.

Tumor-associated circulating miRNAs are derived from tumor cell death and lyses or from active secretion by the tumor [44]. However, a significant proportion originates from immune cells in the blood and the tumor microenvironment or from other organs, potentially representing the host’s response to the presence of the tumor [44]. In NSCLC, distinct deregulated expression profiles of circulating miRNAs have been previously associated with disease progression, prognosis, or drug resistance, thus reflecting distinct genes and pathways [27]. It is however debated whether circulating miRNAs represent simple disease by-products indicative of these processes or whether they actively participate in their regulation [25,45,46]. Circulating miRNAs have indeed been suggested to modulate tumor biology by altering the cellular transcriptome of recipient tumor cells in a paracrine fashion or by delivering signals in distant sites [25,45]. Considering the high tissue specificity of miRNAs [46], the possibility of circulating cancer-associated miRNAs being secreted from blood immune cells to promote or inhibit cancer cell proliferation, invasion, apoptosis, and antitumor immune response by delivering signals to recipient cells cannot be excluded [44]. Thus, miR-21, miR-155, and miR-181a have been involved in both immune response and cancer [47,48,49], indicating that deranged expression of these miRNAs may represent an important link between antitumor immunity and cancer progression [44]. Interestingly, we have previously shown that high circulating miR-21 was associated with disease relapse and shorter disease-free and overall survival in patients with early breast cancer [50].

To uncover potential genes and pathways that could be regulated by the investigated circulating miRNAs, we performed bioinformatics analysis revealing that miR-128 and miR-155 shared 26 target genes in pathways related to response to hypoxia. Further focused analysis of a 26 hypoxia-related gene set revealed additional pathways associated with vasculature development, angiogenesis, apoptosis, and cell death.

Tumor hypoxia has been correlated with tumor aggressiveness and metastasis as well as with adverse outcome in several tumor types [51], suggesting that these miRNAs may promote tumor progression through the regulation of lung cancer cell response to hypoxic states. Interestingly, significant preclinical evidence exists on the role of hypoxia in enhancing DNA damage and mutagenesis as well as in the functional impairment of key genes in DNA repair pathways, ultimately resulting in genomic instability and tumor progression [52]. Moreover, alterations in cell type or tissue-specific miRNA profiles have been reported in response to hypoxia, which then modulates the expression of key components of DNA repair pathways [53,54]. Thus, hypoxia-induced miR-155 has been shown to promote radioresistance [55], whereas in another work, it was implicated in DNA damage response by targeting mutL homolog 1 (MLH1) and mutS homolog 2 (MSH2) involved in DNA mismatch repair [56].

Bioinformatics analysis revealed that hypoxia-inducible factor-1a (HIF-1a), a central regulator of the transcriptional response to hypoxia, was amongst the 26 hypoxia-related gene set of common targets of miR-128 and miR-155. HIF-1a induces miR-155 expression, which in turn targets HIF-1a mRNA, suggesting that miR-155 is a component of a HIF-1α regulatory network during hypoxia [57]. Accordingly, in another study, hypoxia led to vascular endothelial growth factor (VEGF)-induced miR-155 expression that regulated HIF-1a expression and enforced endothelial cell maturation and angiogenesis [58]. In accordance to the above studies, integrated function analysis implicated miR-155 in the regulation of HIF-1a expression.

In the current study, we carefully considered pre-analytical and analytical variables that may influence miRNA detection and quantification; however, methodological approaches for circulating biomarkers need to be validated and standardized across laboratories [59,60]. It should be also noted that our findings regarding the prognostic role of the investigated miRNAs are derived from a relatively small group of patients and lack validation in an independent patient cohort. Identification and further analyses of miRNA protein-coding gene targets relied exclusively on prior knowledge as documented in a reference database [12]. Nevertheless, one should take note of the limitations of such an analysis due to the large numbers of reported targets (even when restricting to cancerous datasets) that are highly likely to reflect the miRNA regulatory potential in variable environments [61]. On the other hand, the significant gene and functional overlaps that we found among the targets of miR-128 and miR-155 suggest that our findings are rather robust and reflect a genuine functional link between the two miRNAs, a link that appears to be quite strong in functions related to hypoxia. Last but not least, out of the 26 common genes targets identified with highly stringent criteria, 23 (>88%) were shown to carry gene regulatory function. This abundance of transcriptional regulators, revealed in our network analysis, is suggestive of a possible regulatory circuitry, in which mir-155 and mir-128 are likely to be key players.

In summary, our results suggest for the first time that pretreatment levels of circulating miR-128 and miR-155 could serve as promising prognostic biomarkers in NSCLC patients treated with first-line platinum-based doublets. Furthermore, using a bioinformatics approach, we showed that these miRNAs are involved in major biological processes in cancer and we highlighted a link between hypoxia, DNA repair, and chemoresistance. Since extracellular miRNAs represent a means of cell–cell communication [25], miR-128 and miR-155 may promote these effects in recipient cells. In addition, although hypoxia-targeted therapy trials in NSCLC have not as yet been translated into patient benefit, if the investigated miRNAs are indeed functional, they could be further exploited therapeutically, taking into account potential histology-driven differences.

## 4. Materials and Methods

### 4.1. Patients’ Characteristics and Sample Collection

Two-hundred-eighteen patients with recurrent or metastatic NSCLC treated with first-line platinum-based chemotherapy at the Department of Medical Oncology, University Hospital of Heraklion (Crete, Greece) between 2009 and 2013 and available plasma were identified from the clinic records (Figure 1). Blood was obtained during routine blood collection performed before the administration of chemotherapy. Plasma samples were also collected from 19 healthy blood donors (control group) during the procedure of volunteer blood donation performed in the Blood Bank Department of the University General Hospital of Heraklion. The median age of healthy individuals was 63 years (range 55–68); 16 were males, and 3 were females. All patients and healthy donors had signed an informed consent to participate in the study, which was approved by the Ethics and Scientific Committee of the University Hospital of Heraklion (ID 5532/8-6-2016; Crete, Greece). Clinical characteristics and follow-up information for each patient were prospectively collected. Response to treatment was evaluated by CT scans (or MRI scans when indicated) using RECIST 1.1 criteria [29]. Peripheral blood from healthy donors and patients was drawn early in the morning and was collected in ethylenediaminetetraacetic acid EDTA tubes. In patients, blood samples were obtained the same day before starting chemotherapy. Plasma was subsequently isolated within 2 h by centrifugation in 2500 rpm for 15 min at 4 °C, followed by a second centrifugation in 2000× *g* for 15 min at 4 °C to remove cellular debris. Samples were kept in aliquots at −80 °C until further use. Plasma samples presenting a change of colour to pink (n = 16), suggesting the presence of hemolysis, were not processed for further analysis (Figure 1).

### 4.2. RNA Isolation

Total RNA was isolated using Trizol LS (Ambion, Life Technologies) from 400 μL of plasma as described previously [62]. Briefly, plasma was thawed on ice and centrifuged to remove cellular debris. After denaturation, 25 fmoles of the synthetic *C. elegans* miRNA cel-miR-39 (Qiagen GmbH, Hilden, Germany) was added to each sample as an exogenous control. Aqueous phase was separated by the addition of chloroform followed by centrifugation and equal volume of 700 μL of supernatant; each sample was precipitated by adding 0.7 volumes of isopropanol and 1 μL of glycogen (final concentration, 13 μg/mL) (QIAGEN). RNA pellet was resuspended in 50 μL RNAse-free water. RNA from all samples was kept at −80 °C until further use in the subsequent real-time qPCR.

### 4.3. Quantitative Real-Time PCR Analysis and miRNA Expression

Reverse transcription and RT-qPCR was performed using TaqMan technology according to manufacturer’s instructions and as previously described [24,62]. cDNA synthesis was performed using miRNA specific stem-loop primers (assays ID for each miRNA are provided in Table S1; Applied Biosystmes, Foster City, CA, USA) in a 5-μL reaction and next diluted at 30 μL. Each miRNA was assessed by RT-qPCR in a ViiA 7 Real-Time PCR System (Applied Biosystems, Foster City, CA, USA). All the assays were performed in triplicates. Appropriate negative controls were used in both cDNA synthesis and RT-qPCR reactions, where RNA input was replaced by H_2_O and no template control was used, respectively. The fold change (log_10_) in each miRNA expression relative to the reference gene U6 snRNA was calculated by the 2^−ΔCt^ method.

We choose U6 snRNA as a reference gene since it was stably and reproducibly expressed among patients and healthy donors (Figure S1; Mann–Whitney test, *p* = 0.123). The expression levels (log_10_) of each target miRNA relative to miRNA expressed in healthy controls was calculated by the 2^−ΔΔCt^ method [63]. The suitability of U6 snRNA for the quantification of circulating miRNA levels was further supported by the fact that the ΔCt between target miRNAs and U6 snRNA was low, demonstrating a similar range of expression. This is very important in order to measure both target and reference genes in one sample dilution. As mentioned above, besides the macroscopic examination, samples were also evaluated for the presence of hemolysis by measuring miR-451 and miR-23a expression levels [64]. Samples contaminated with red blood cells were not processed for further miRNA expression analysis (n = 24; Figure 1). Samples with mean Ct > 35 or not amplified for target miRNAs (n = 5) and samples with mean Ct > 22 or Ct < 20 of cel-miR-39 (n = 5), suggesting inefficient RNA extraction, were excluded from the statistical analysis (Figure 1). Finally, samples with mean Ct > 33 or Ct < 30 of U6 snRNA (n = 7) were also excluded from the statistical analysis (Figure 1).

### 4.4. miRNA Gene Target and Pathway Enrichment Analysis

We obtained the protein-coding gene targets for the miRNAs under study from TarBase [65]. For each miRNA, we retrieved the full set of human protein-coding genes that were found associated in cancerous tissues. Large numbers of gene targets are reported for both hsa-miR-155 (4259 genes) and hsa-miR-128 (2329 genes). Out of those, there were 860 genes in common, which constituted a highly significant degree of overlap (*p*-value of a hypergeometric test < 10^−77^). We then proceeded in a functional enrichment analysis for the gene targets of each miRNA separately as well as for the subset of common targets. We used gProfiler [66] to calculate the enrichment significance for Gene Ontology (GO) and for Biological Pathways (BP) as compiled by the Kyoto Encyclopedia of Genes and Genomes (KEGG), BioCarta, and Reactome Databases. In order to avoid reporting biases for very general or very confined functional categories, we filtered out enrichments for terms that contained ≤10 or ≥500 genes. The top 10 most enriched, nonredundant biological processes ranked according to an adjusted *p*-value are shown in Table 5.

Protein–protein interaction (PPI) analysis was performed with the STRING database (STRING-DB; https://string-db.org/) [67]. Clustering was performed with Markov cluster (MCL) as implemented by STRING-DB.

### 4.5. Statistical Analysis

Statistical package of the social sciences (SPSS) software package, version 22.0 (SPSS Inc. Chicago IL, USA) was used to perform statistical analysis. Patients with miRNA expression above or equal to the median values were characterized as having high expression, whereas patients with miRNA expression below the median were characterized as having low expression. Correlations of expression between the different miRNAs were performed by Spearmans’ test. The chi-squared test was used to estimate associations between miRNA expression and clinicopathological characteristics. Differential expression was examined by Mann–Whitney test. The associations between circulating miRNA expression levels and PFS or OS were assessed by the Kaplan Meier method, log rank test (Mantel–Cox), and Cox proportional hazard regression models. PFS and OS were calculated from the start of treatment until the date of the first documented disease progression or death and last follow-up, respectively.

Statistical significance was set at *p* < 0.05 (two-sided test). This report is written according to the Reporting recommendations for tumor marker prognostic studies (REMARK criteria) [68].

## 5. Conclusions

In summary, we here demonstrate that the expression levels of DDR-related miRNAs evaluated in the plasma hold significant prognostic implications in NSCLC patients treated with first-line platinum-based chemotherapy, further supporting the role of circulating miRNAs as important cancer biomarkers. Bioinformatics analysis suggests that the adverse prognostic role of miR-128 and miR-155 could be related to their functional involvement in pathways related to response of recipient cells to hypoxia resulting in altered DNA damage response, chemoresistance, and angiogenesis. Further studies are needed to investigate the potential functional role of these miRNAs in an effort to exploit their therapeutic potential.

## Figures and Tables

**Table 1:** Patients’ characteristics.

	All Patients		SqCC		non-SqCC		
Characteristic	*N*	%	*N*	%	*N*	%	*p* Value
Number of patients	128		41	32	87	68	
Gender							0.002 ^a^
Male	111	87	41	100	70	80	
Female	17	13			17	20	
Age (years)							0.138 ^a^
median (range)	65 (37–88)		66 (46–88)		64 (37–82)		
ECOG PS							0.172 ^a^
0	31	24	11	27	20	23	
1	79	62	22	54	57	66	
2	18	14	8	19	10	11	
Stage at diagnosis ^b^							0.001 ^a^
II	1	1	1	2			
III	4	3	4	10			
IV	123	96	36	88	87	100	
Histology							ns ^a^
Adenocarcinoma	79	62					
Squamous	41	32					
Other	8	6					
Number of metastatic sites							0.037 ^a^
0	16	13	6	15	10	12	
1	50	39	22	54	29	33	
2	34	26	9	22	25	29	
≥3	28	22	4	9	23	26	
Prior therapy ^c^							
Palliative RT	26	20	5	12	21	24	
Radical thoracic RT for localized disease	3	2	3	7			
Chemotherapy regimens							
CDDP/TXT	47	37	19	46	28	32	
CDDP/GEM	36	28	21	51	15	17	
CDDP/PEM	45	35	1	3	44	51	
Response ^c^							0.567 ^a^
PR	33	26	13	32	20	23	
SD	50	39	14	34	36	41	
PD	45	35	14	34	31	36	

SqCC, squamous cell carcinoma; non-SqCC, non-squamous cell carcinoma; ECOG PS, Eastern Cooperative Oncology Group Performance Status; RT, radiotherapy; CDDP, cis-diamminedichloridoplatinum; TXT, taxotere; GEM, gemcitabine; PEM, pemetrexed; PR, partial response; SD, stable disease; PD, progressive disease; ns, non-significant. ^a^ Pearson’s chi-squared test for comparison between patients with SqCC and non-SqCC. ^b^ The patient with stage II disease at diagnosis had been treated with definite radiotherapy as initial therapy. Two out of four patients with stage III lung cancer at diagnosis had received prior definitive chemoradiotherapy. The above three patients subsequently received first-line platinum doublets on disease progression. The remaining 2 patients that presented with stage III disease were not amenable to definite radiotherapy and were treated with platinum doublet chemotherapy as single treatment modality. ^c^ Response to treatment was assessed according to The Response Evaluation Criteria in Solid Tumors (RECIST 1.1 criteria) [29].

**Table 2:** Spearman’s correlation among miRNAs.

miRNA	miR-21	miR-128	miR-155	miR-181a
miR-21	1			
miR-128	0.853 **	1		
miR-155	0.829 **	0.855 **	1	
miR-181a	0.896 **	0.929 **	0.886 **	1

** Spearman’s Rho, *p* < 0.001.

**Table 3:** Univariate and multivariate Cox regression analysis for overall survival in NSCLC patients.

**Univariate Analysis**		
**Cox Regression**	**HR (95% CI)**	***p* Value**
Age (<65 vs. ≥65)	1.232 (0.853–1.780)	0.266
Gender (male vs. female)	1.425 (0.826–2.458)	0.203
ECOG PS (2 vs. 0–1)	2.465 (1.473–4.124)	0.001 *
Stage at diagnosis (IV vs. others)	1.698 (0.625–4.612)	0.299
Histology (SqCC vs. non-SqCC)	1.067 (0.734–1.552)	0.733
Number of metastatic sites (≥2 vs. 0–1)	1.686 (1.166–2.437)	0.006 *
miR-21 expression (high vs. low)	1.322 (0.918–1.903)	0.134
miR-128 expression (high vs. low)	1.499 (1.041–2.160)	0.030 *
miR-155 expression (high vs. low)	1.481 (1.026–2.137)	0.036 *
miR-181a expression (high vs. low)	1.235 (0.858–1.779)	0.257
**Multivariate Analysis**		
**Cox Regression**	**HR (95% CI)**	***p* Value**
ECOG PS (2 vs. 0–1)	2.199 (1.304–3.708)	0.003 *
Number of metastatic sites (≥2 vs. 0–1)	1.270 (0.852–1.904)	0.237
miR-128 expression (high vs. low)	1.539 (1.054–2.247)	0.026 *
miR-155 expression (high vs. low)	1.143 (0.670–1.951)	0.623

HR, Hazard Ratio; CI, Confidence Intervals; ECOG PS, Eastern Cooperative Oncology Group Performance Status; patients classified into high and low expression groups according to the median value of each miRNA; Cox regression, * *p* < 0.05.

**Table 4:** Univariate and multivariate Cox regression analysis for overall survival in the squamous cell carcinoma (SqCC) subgroup of NSCLC patients.

**Univariate Analysis**		
**Cox Regression**	**HR (95% CI)**	***p* Value**
Age (<65 vs. ≥65)	1.818 (0.931–3.550)	0.08
ECOG PS (2 vs. 0–1)	2.635 (1.149–6.042)	0.022 *
Stage at diagnosis (IV vs. others)	1.698 (0.625–4.612)	0.299
Number of metastatic sites (≥2 vs. 0–1)	1.937 (0.919–4.082)	0.082
miR-21 expression (high vs. low)	2.185 (1.099–4.343)	0.026 *
miR-128 expression (high vs. low)	2.582 (1.230–5.421)	0.012 *
miR-155 expression (high vs. low)	2.860 (1.406–5.819)	0.004 *
miR-181a expression (high vs. low)	2.181 (1.080–4.406)	0.03 *
**Multivariate Analysis**		
**Cox Regression**	**HR (95% CI)**	***p* Value**
ECOG PS (2 vs. 0–1)	1.992 (0.846–4.694)	0.114
miR-21 expression (high vs. low)	1.350 (0.479–3.806)	0.570
miR-128 expression (high vs. low)	2.788 (0.674–11.539)	0.157
miR-155 expression (high vs. low)	2.860 (1.406–5.819)	0.004 *
miR-181a expression (high vs. low)	4.910 (0.267–9.155)	0.284

HR, Hazard Ratio; CI, Confidence Intervals; ECOG PS, Eastern Cooperative Oncology Group Performance Status; patients classified into high and low expression groups according to the median value of each miRNA; Cox regression, * *p* < 0.05.

**Table 5:** Top 10 most enriched biological processes (GO:BP) for the protein-coding gene targets of hsa-miR-128, hsa-miR-155, and their common targets ranked according to an adjusted *p*-value of enrichment.

Biological Process (GO:BP)	Adjusted *p*-Value
**hsa-miR-128 targets**	
dendrite development	1.92 × 10^−05^
regulation of cytoskeleton organization	4.83 × 10^−05^
regulation of protein serine/threonine kinase activity	6.65 × 10^−05^
proteasomal protein catabolic process	0.000311
positive regulation of cellular catabolic process	0.000512
peptidyl-serine modification	0.000762
regulation of cell morphogenesis	0.000788
proteasome-mediated ubiquitin-dependent protein catabolic process	0.000811
cellular response to decreased oxygen levels	0.000922
positive regulation of cell migration	0.00106
**hsa-miR-155 targets**	
regulation of binding	4.76 × 10^−15^
regulation of protein catabolic process	1.55 × 10^−12^
proteasomal protein catabolic process	1.35 × 10^−09^
response to oxidative stress	3.07 × 10^−09^
cellular response to external stimulus	3.94 × 10^−09^
regulation of mitotic cell cycle phase transition	6.31 × 10^−09^
regulation of protein binding	6.90 × 10^−09^
regulation of protein serine/threonine kinase activity	1.23 × 10^−08^
G2/M transition of mitotic cell cycle	4.17 × 10^−08^
Cell–cell signaling by wnt	6.37 × 10^−08^
**common targets (hsa-miR-155/hsa-miR-128)**	
cellular response to hypoxia	5.41 × 10^−05^
extrinsic apoptotic signaling pathway	0.00421
regulation of cellular amide metabolic process	0.00697
regulation of transcription from RNA polymerase II promoter in response to stress	0.00954
response to oxygen levels	0.0121
regulation of extrinsic apoptotic signaling pathway	0.0137
proteasomal protein catabolic process	0.014
response to decreased oxygen levels	0.019
regulation of protein catabolic process	0.0198
tissue remodeling	0.0203

GO, gene ontology; BP, biological pathways; adjusted *p*-value by Benjamini–Hochberg, false discovery rate (FDR) ≤ 5%.

**Table 6:** Twenty-six genes associated to hypoxia and predicted to be targeted by both miR-128 and miR-155.

Gene Name	Gene Description	ENSEMBL ID
AJUBA	ajuba LIM protein	ENSG00000129474
ANKRD1	ankyrin repeat domain 1	ENSG00000148677
BACH1	BTB domain and CNC homolog 1	ENSG00000156273
BRIP1	BRCA1 interacting protein C-terminal helicase 1	ENSG00000136492
CARD16	caspase recruitment domain family member 16	ENSG00000204397
CAV1	caveolin 1	ENSG00000105974
CITED2	Cbp/p300 interacting transactivator with Glu/Asp rich carboxy-terminal domain 2	ENSG00000164442
DDAH1	dimethylarginine dimethylaminohydrolase 1	ENSG00000153904
EGR1	early growth response 1	ENSG00000120738
ENO1	enolase 1	ENSG00000074800
EPAS1	endothelial PAS domain protein 1	ENSG00000116016
GNB1	G protein subunit beta 1	ENSG00000078369
HIF1A	hypoxia inducible factor 1 subunit alpha	ENSG00000100644
HMOX1	heme oxygenase 1	ENSG00000100292
ICAM1	intercellular adhesion molecule 1	ENSG00000090339
KCNK3	potassium two pore domain channel subfamily K member 3	ENSG00000171303
NDNF	neuron-derived neurotrophic factor	ENSG00000173376
NDRG1	N-myc downstream regulated 1	ENSG00000104419
NFE2L2	nuclear factor, erythroid 2 like 2	ENSG00000116044
PDK1	pyruvate dehydrogenase kinase 1	ENSG00000152256
PTGS2	prostaglandin-endoperoxide synthase 2	ENSG00000073756
RBPJ	recombination signal binding protein for immunoglobulin kappa J region	ENSG00000168214
RORA	RAR-related orphan receptor A	ENSG00000069667
TMBIM6	transmembrane BAX inhibitor motif containing 6	ENSG00000139644
VEGFA	vascular endothelial growth factor A	ENSG00000112715
ZFP36L1	ZFP36 ring finger protein like 1	ENSG00000185650

## Supplementary Materials

The following are available online at https://www.mdpi.com/2072-6694/12/5/1282/s1, Figure S1: U6 snRNA expression levels between NSCLC patients and healthy donors, Table S1: Assay ID for each miRNA used in the study.
