# Supplementary material for: Circulating MicroRNAs Regulating DNA Damage Response and Responsiveness to Cisplatin in the Prognosis of Patients with Non-Small Cell Lung Cancer Treated with First-Line Platinum Chemotherapy
Source: Cancers (Basel). 2020 May 19;12(5):1282. doi: 10.3390/cancers12051282 (PMC7281609; doi:10.3390/cancers12051282)
Supplement: Supplementary file 1 [file cancers-12-01282-s001.pdf]

Supplementary Material: Circulating MicroRNAs Regulating DNA Damage Response and Responsiveness to Cisplatin in the Prognosis of Patients with Non-Small Cell Lung Cancer Treated with First-Line Platinum Chemotherapy

Chara Papadaki, Alexia Monastirioti, Konstantinos Rounis, Dimitrios Makrakis, Konstantinos Kalbakis, Christofors Nikolaou, Dimitrios Mavroudis and Sofia Agelaki

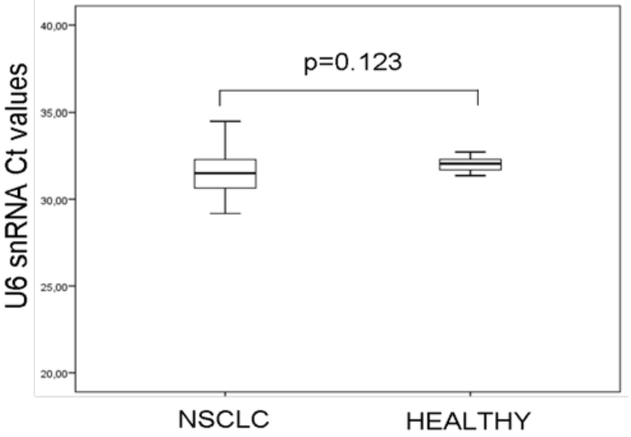

Figure S1. U6 snRNA expression levels between NSCLC patients and healthy donors. Mann-Whitney test was used to determine statistically significant differences and the results were displayed on box plots. Horizontal line depicts the median Ct value, whereas the length of the boxes is the interquartile range that represents values between the 75th and 25th percentiles of individual Ct values. *P* values are shown.

Table S1. Assay ID for each miRNA used in the study.

Name	Assay ID
has-miR-21-5p	000397
has-miR-128a-3p	002216
has-miR-155-5p	002623
has-miR-181A-5p	000480
U6 snRNA	001973
cel-miR-39-3p	000200
